# *In vitro* cytotoxicity of *Artemisia vulgaris* L. essential oil is mediated by a mitochondria-dependent apoptosis in HL-60 leukemic cell line

**DOI:** 10.1186/1472-6882-14-226

**Published:** 2014-07-07

**Authors:** Ayman M Saleh, Ahmad Aljada, Syed AA Rizvi, Amre Nasr, Ahmed S Alaskar, Jack D Williams

**Affiliations:** 1 College of Medicine, King Saud Bin Abdulaziz University for Health Sciences (KSAU-HS), Riyadh, Saudi Arabia; 2 King Abdullah International Medical Research Center (KAIMRC), P.O. Box: 3660, Riyadh 11481, Saudi Arabia; 3 College of Pharmacy, Nova Southeastern University (NSU), Fort Lauderdale, Florida, USA; 4 Division of Adult Hematology & HSCT, Department of Oncology, King Abdulaziz Medical City, P.O. Box 22490, Riyadh 11426, Saudi Arabia; 5 Department of Chemistry and Biochemistry, Mercyhurst University, Erie, PA, USA

**Keywords:** *Artemisia vulgaris*, Essential oil, Cytotoxicity, Apoptosis, Mitochondria, Caspases, Bcl-2 family

## Abstract

**Background:**

The essential oil (EO) of *Artemisia vulgaris* L. has been traditionally used worldwide for treating a large number of diseases. Although major components in *A. vulgaris* EO have been shown to inhibit growth of different cancer cells, as pure compounds or part of other plants extracted oil, no information is known about its anti-proliferative activities. Therefore, the current investigation has evaluated the toxicity of the plant extracted oil from buds (AVO-b) and leaves (AVO-l) and characterized their growth inhibitory effects on cancer cells.

**Methods:**

AVO-b and AVO-l from *A. vulgaris* L. were extracted by hydrodistillation, and their effect on the viability of human HL-60 promyelocytic leukemia and various other cancer cell lines was tested using MTT assay. Flow cytometric analysis of apoptosis, DNA fragmentation assay, caspases enzymatic activities and Western blotting were used to determine the apoptotic pathway triggered by their action on HL-60 cells.

**Results:**

Low concentrations of AVO-b and AVO-l inhibited the growth of HL-60 cells in a dose- and time-dependent manner. Employing flow cytometric, DNA fragmentation and caspase activation analyses, demonstrated that the cytotoxic effect of the oils is mediated by a caspase-dependent apoptosis. Kinetic studies in the presence and absence specific caspase inhibitors showed that activation of caspase-8 was dependent and subsequent to the activation of caspases-9 and -3. In addition, the essential oil caused a disruption of the mitochondrial transmembrane potential (ΔΨ_m_), increased the release of cytochrome *c* to the cytosol, and altered the expression of certain members of Bcl-2 family (Bcl-2, Bax and Bid), Apaf-1 and XIAP. Interestingly, low doses of AVO-b and AVO-1 also induced apoptosis in various cancer cell lines, but not in noncancerous cells.

**Conclusions:**

The results demonstrate that the EO-induced apoptosis in HL-60 cells is mediated by caspase-dependent pathways, involving caspases-3, -9, and -8, which are initiated by Bcl-2/Bax/Bid-dependent loss of ΔΨ_m_ leading to release of cytochrome *c* to the cytoplasm to activate the caspase cascade. The finding that AVO-b and AVO-l are more efficient to induce apoptosis in different cancer cell lines than noncancerous cells, suggests that *A. vulgaris* might be a promising source for new anticancer agents.

## Background

Cancer is one of the leading causes of death worldwide [1]. Although the current anticancer drugs continue to play a major role in cancer treatment, there are still many types of cancers do not respond effectively to the available therapeutics. Hence, there is an impetus to identify, develop and test more potent anticancer therapeutics [2].

One of the key methods of successful chemotherapeutics is their ability to trigger death of cancer cells through apoptosis [3,4]. These anticancer agents are able to inhibit cell growth by acting on two principal signaling pathways of apoptosis. One pathway (the extrinsic/receptor mediated) involves ligation of death receptors, such as Fas receptor (Fas) and other members of the tumor-necrosis factor (TNF) receptor family, resulting in recruitment and activation of caspase-8 [5,6]. The second pathway (intrinsic/mitochondria dependent) is stimulated by the release of cytochrome *c* from the mitochondrial intermembrane space to the cytosol allowing activation of caspase-9 [7,8]. Following activation of the initiator caspase-8 or -9, the two pathways converge on the activation of caspase-3, which finally execute the death process by cleaving various vital substrates required for cell survival and maintaining the integrity of the genomic DNA [5]. Although these pathways are distinct from each other, they cross-communicate (i.e. activation of one pathway triggers activation of the other) to amplify the apoptotic signal [9].

*Artemisia vulgaris* L. (commonly known as mugwort) belongs to the Asteraceae family of plants, which consists of more than 500 species that are globally distributed. The plant is traditionally used to treat a wide range of conditions, including gastrointestinal disorders, headaches, nose bleeds, muscle spasms, epilepsy, circulatory problems, menopausal and menstrual complaints, fever, rheumatism, asthma, gout, infertility, contact dermatitis, bacterial infections, inflammation, malaria and worm infestations [10,11].

Recently, there has been increasing interest in the use of essential oils as medicinal agents, because they have been found to have anticancer potentials through induction of apoptosis in various cancer cell lines of hematological and solid tumor origins [12,13]. There is considerable evidence showing that the active compounds in the essential oils of different *Artemisia* species are responsible for their anti-proliferative effect on cancer cells [14-19]. Although there is no available scientific data on the cytotoxic and apoptosis inducing effects of *A. vulgaris* essential oil, previous evidence indicate that the aqueous methanol extract from dry leaves of this plant is cytotoxic to the human hepatocellular carcinoma cell line HepG2 that is suggested to be mediated by apoptosis [20]. Aqueous extracts from *A. vulgaris* have been also reported to induce apoptosis in prostate, breast and colon cancer cell lines [21]. In addition, extracts from *A. vulgaris* have been shown to sensitize MDA-MB-231 and MDA-MB-468 breast cancer cells to TRAIL [22]. In a recent study, we have isolated the essential oils from *A. vulgaris* aerial parts (leaves and buds) and identified its chemical composition using gas chromatography (GC)/mass spectrometry (MS) analyses [23]. Our results have identified 22 compounds in *A. vulgaris* L. essential oils which majorly include germacrene D (25%), caryophyllene (20%), alpha-zingiberene (15%) and borneol (11%) in the leaf oil, while the buds are rich in 1,8-cineole (32%), camphor (16%), borneol (9%), and caryophyllene (5%). Major components of the oil such as caryophyllene [24], alpha-zingiberene [25], borneol [26] and ar-curcumene [27] all have been reported to induce apoptosis in different human cancer cell lines, as purified compounds or as part of essential oil isolated from other plants.

In this study, we have examined whether or not the essential oil isolated from the aerial parts of *A. vulgaris* L. induces apoptosis in the human acute myelogenous leukemia cell line HL-60. This report has also investigated the possible mechanism (s) of apoptosis triggered by the essential oil. The results demonstrate, for the first time, that low doses of essential oil from *A. vulgaris* L. induce apoptosis in the HL-60 cells through a mitochondria and caspase-dependent mechanisms. In addition to the effect on HL-60, low concentrations of the essential oils from leaves and buds were able to induce apoptosis in various other cancer cell lines, such as Jurkat, K562, MCF-7, HepG2, PC-3 and HeLa, but lack substantial cytotoxicity for normal non-malignant cells such as BJ and HEK-293 V79-4 cells at the same doses.

## Methods

### Reagents

4,5-dimethylthiazol-2yl)-2,5-diphenyltetrazolium bromide (MTT), JC-1 (5,5′,6,6′-tetrachloro-1,1′,3,3′-tetraethylbenzimidazolocarbocyanine iodide), protease inhibitors (PMSF, pepstatin A, leupeptin, and aprotinin) and etoposide [4′-Demethylepipodophyllotoxin 9-(4,6-O-ethylidene-β-D-glucopyranoside)] were acquired from Sigma Aldrich (St Louis, MO, USA). Cell culture media (RPMI 1640 and DMEM), penicillin-streptomycin, and fetal bovine serum (FBS) were purchased from Invitrogen (Invitrogen, Carlsbad, CA, USA). The colorimetric tetrapeptide substrates for caspase-8 (Ac-IETD-*p*NA), caspase-9 (Ac-LEHD-*p*NA) and caspase-3 (Ac-DEVD-*p*NA) were purchased from Calbiochem (San Diego, CA). Caspase-8 inhibitor (zIETD-fmk), caspase-9 inhibitor (zLEHD-fmk), caspase-3 inhibitor (zDEVD-fmk), and the general caspase inhibitor (zVAD-fmk) were obtained from Alexis Corporation (Switzerland). The polyclonal or monoclonal antibodies against caspase-8, cytochrome *c*, Apaf-1, XIAP, α-tubulin, β-actin, VDAC, Bax, Bak, Bcl-2 and Bcl-xL proteins were obtained from Santa Cruz Biotechnology (CA, USA), while the ones for caspase-9 and caspase-3 are from Stressgen Biotechnologies (Victoria, British Columbia, Canada).

### Plant material

Fresh *Artemisia vulgaris* L. plants were collected during July 2010 through August of 2010 from Erie, Pennsylvania in the United States of America. The plant identity was confirmed by Dr. Marlene Cross, Department of Biology, Mercyhurst University, and a reference voucher specimen (Reference number: TREC He- 00818) was deposited in the Herbarium of the Tom Ridge Center, Erie, Pennsylvania [23]. In addition, the identity of the same plant used in this study was further confirmed using DNA sequencing as recently described by [28]. The leaves and buds (before flowering) were washed, separated and dried before further analysis.

### Isolation of essential oils

We have previously described the isolation and chemical composition of essential oils from *A. vulgaris* aerial parts [23]. Briefly, 334 g of macerated *A. vulgaris* buds were introduced into a two liter round bottom flask and soaked in 1.68 L double distilled water. The flask material was subjected to hydrodistillation using a Clevenger-type apparatus. Approximately, 966 mg of the bud’s essential oil (AVO-b) was collected after four hours. A similar procedure was followed to extract the essential oil from the plant fresh leaves (600 g); which yielded 560 mg of light yellow oil (AVO-l). Both samples of AVO-l and AVO-b were aliquoted in small glass tubes (50 μg each) and stored at -20°C, but allowed to warm to room temperature before addition to cultured cells. The density of AVO-l was 0.909 g/mL and 0.947 g/mL for AVO-b. Proper dilutions of the oils were performed using a 25% DMSO in phosphate buffered saline (PBS).

### Cell culture conditions

The human acute promyelocytic leukemia HL-60 (ATCC® CCL-240™), Jurkat (human acute T lymphocytic leukemia, ATCC® TIB-152™) and K-562 (human chronic myelogenous leukemia, ATCC® CCL-243™) suspension cells were maintained in RPMI-1640, while HepG2 (human hepatocellular carcinoma, ATCC® HB-8065™), MCF7 (human breast adenocarcinoma, ATCC® HTB-22™), PC-3 (human prostate adenocarcinoma, ATCC® CRL-1435™), HeLa (human cervical Adenocarcinoma, ATCC® CCL-2™^)^, HEK-293 (human embryonic kidney, ATCC® CRL-1573 ™), BJ (human skin fibroblast, ATCC® CRL-2522™ ) and V79-4 (Chinese hamster lung fibroblast, ATCC® CCL-93™) adherent cells were cultured in DMEM medium supplemented with 10% (V/V) heat inactivated FBS, penicillin G (100U/mL) and streptomycin (100 mg/mL) at 37^∘^C in a 5% CO2 humidified incubator. The media were changed 2–3 days and subcultured when the cell population density reached to 70–80% confluence. Cells were seeded at an appropriate density according to each experimental design.

### Cell viability (cytotoxicity) assay

The cell viability was assessed using the MTT assay [29]. Briefly, 5 × 10^4^ of HL-60, in a 100 μL of RPMI-1640 medium were seeded in each well of a 96 well-plate. After 24 h, increasing concentrations of AVO-l or AVO-b (0.0 to 2.0 μg/mL) were added and incubated for 24 h in a 5% CO_2_-cell culture incubator. Alternatively, cultured cells were incubated with 1.0 μg/mL of AVO-l or AVO-b for different time-points (0, 4, 8, 12, 24, 36 and 48 h) before analysis. At the end of incubation period with the oil, 50 μL of MTT (2 mg/mL stock solution) were added and the plates and incubated for an additional 4 h. The resulting MTT-formazan product was dissolved by addition of 100 μL of DMSO and incubation was continued for additional 4 h. The amount of formazan was determined by measuring the absorbance at 570 nm using an E Max Precision Microplate reader (Molecular Devices, Sunnyvale, CL, USA). The concentration of the essential oil leading to 50% inhibition of viability (IC_50_) was calculated using non-linear regression analysis of GraphPad Prism 5 software. When caspase inhibitors were used, HL-60 cells were incubated with either 35.0 μM of Caspase-8 inhibitor (zIETD-fmk), caspase-9 inhibitor (zLEHD-fmk), caspase-3 inhibitor (zDEVD-fmk), or 50.0 μM of the general caspase inhibitor (zVAD-fmk) for 6 h prior to addition of the extracted oil. In all experiments, the final concentration of DMSO was ≤ 0.05%. Similar concentrations of DMSO were added to the control of untreated cells, and showed no effect on cell viability, when compared to the viability of cells without DMSO. Under similar conditions, HL-60 cells were treated with 4.0 or 8.0 μM etoposide as a positive control to validate the results of MTT assay.

### Analysis of apoptosis by flow cytometry

The percentage of cells undergoing apoptosis after treatment with increasing concentrations of *A. vulgaris* oil essential oil (AVO) was determined by using the Muse™ Annexin V & Dead Cell Assay kit (EMD Millipore Bioscience, Darmstadt, Germany) according to the manufacturer’s protocol. The kit utilizes a fluorescent dye (FITC) conjugated to Annexin-V to detect phosphatidylserine (PS) on the external membrane of apoptotic cells and 7-AAD (7-amino-actinomycin D) as a dead cells marker. 7-AAD is excluded from living healthy cells, as well as early apoptotic cells. Percentages of cells in early (Annexin-V^+ve^/7-AAD^-ve^) and late stages of apoptosis (Annexin-V^+ve^/7-AAD^+ve^) were determined by a flow cytometer-based instrument (Muse™ Cell Analyzer, EMD Millipore Bioscience). In all experiments, the solvent DMSO concentration was ≤ 0.05%. Similar concentrations of DMSO were added to the control of untreated cells, and showed no effect on triggering apoptosis, when compared to cultured HL-60 cells in the absence of DMSO. HL-60 cells were also treated with 8 μM etoposide as a positive control to validate the apoptosis results.

### Detection of DNA fragmentation

DNA fragmentation was analyzed using agarose gel electrophoresis. Genomic DNA was prepared using the method previously described in details by [30,31] with slight modifications. After treating 2 × 10^6^ cultured HL-60 cells with increasing concentrations of AVO-l or AVO-b for 24 h, cells were harvested by centrifugation at 500 *g* for 5 min, and washed twice with 1.0 mL PBS. The pelleted cells were subsequently lysed in a buffer containing 50 mM Tris–HCl, pH 8.0, 10 mM EDTA, 0.5% SDS and 0.5 mg/mL proteinase K at room temperature for 30 min. The mixtures was treated with RNase A (25 mg/mL) at 37°C for 30 min. The DNA was extracted and purified sequentially with phenol: chloroform (1:1) and chloroform followed by precipitation in 100% ice-cold ethanol. The samples were then air dried and resuspended in TE buffer (10 mM Tris HCl, 1 mM EDTA, pH 7.5). 5.0 μg of each of DNA samples were resolved by electrophoresis for 4 h on a 1.5% agarose gel and stained with ethidium bromide and the banding patterns were visualized with the Foto/Eclipse UV transilluminator. The presence of late apoptosis events were indicated by the appearance of a ladder of oligonucleosomal DNA fragments which are approximately 180–200 bp multiples. A DNA size maker was used as a molecular size standard.

### Assays of caspase-9, -8, and -3 activities

The activities of caspase-9, -8, and -3 in HL-60 cell lysates (30 μg), obtained after treating with the proper concentration of AVO-l or AVO-b for the desired time periods in the presence or absence of the specific caspase inhibitors, were determined spectrophotometricaly at 405 nm using a microtiter plate reader. The assays were performed by incubating the cell lysates with 0.2 mM of the caspase-specific colorimetric tetrapeptide substrates, Ac-LEHD-*p*NA (for caspase-9), Ac-IETD-*p*NA (for caspase-8) or Ac-DEVD-*p*NA (for caspase-3) for 1 h at 37°C as described by [31]. The increase in the absorbance at 405 nm which corresponds to the amount of *p*-nitroaniline (*p*NA) liberated from the peptide substrates was converted into units of enzyme activity using a standard curve generated with free *p*NA. One unit of caspase-3, -8, or -9 activity correspond to the amount of enzyme that will release 1 pmol of *p*NA from 0.2 mM DEVD-*p*NA, IETD-*p*NA or Ac-LEHD-pNA per min, respectively. Lysates from HL-60 cells treated with etoposides were also used in these assays as positive control to validate the enzymatic assays.

### Assessment of changes in mitochondrial transmembrane potential

Measurement of changes in mitochondria membrane potential (ΔΨ_m_) was performed with the fluorescent stain JC-1. This dye accumulates in mitochondria in an MMP (mitochondria membrane potential)-dependent manner, showing red fluorescent JC-1 aggregates (590 nm emissions) at higher MPP. When MMP decreases, JC-1 aggregates disappear from mitochondria and change to green fluorescent JC-1 monomers (535 nm emissions). Therefore, the ratio of the red signal to the green can been used to detect the changes of MMP (ΔΨ_m_) due to depolarization in the early stages of cell death as a result of mitochondrial damage [32,33]. Measurement of mitochondria ΔΨ_m_ in AVO-treated cells was performed as previously described in [34] with some modifications. Briefly, after treating the HL60 cells with AVO-l or AVO-b for different periods of time, cells were incubated at room temperature in the dark with 5 μg/mL JC-1 in HBSS (Hank’s balanced salt solution, Invitrogen, USA) for 30 minutes. The cells were then washed twice with HBSS and fluorescence levels were immediately acquired with excitation and emission wavelengths set at 535 and 590 nm, respectively, for red fluorescence, and 485 and 535 nm, respectively, for green fluorescence. Measurements were performed in a 96-well plate using a fluorometer plate reader (FLUOstar OPTIMA F microplate fluorometer, BMG LABTECH GmbH, Germany). For each sample, the results were calculated as the ratio (red/green) of fluorescence of sample, averaged after the fluorescence values had been corrected for the background. HL-60 cells treated with etoposide were used as positive controls to validate the fluorometry results. In all experiments, the solvent DMSO concentration was ≤ 0.05%. Similar concentrations of DMSO were added to the control of untreated cells, and showed no effect on triggering changes in mitochondria transmembrane potential, when compared to cultured HL-60 cells in the absence of DMSO.

### Isolation of mitochondrial and cytosolic fractions

Mitochondrial and cytosolic fractions from HL-60 cells were prepared by differential centrifugation at 4°C as previously described [35,36]. Briefly, The pellets of AVO-b-treated cells were washed twice with PBS (pH 7.4) and resuspended in a 5 volumes of buffered-medium containing 70 mM sucrose, 220 mM mannitol, 2.5 mM Hepes, pH 7.4, 2 mM EDTA, 1 mM DTT, and 0.1 mM PMSF. Subsequently, the cells were homogenized in a glass Dounce homogenizer (20 strokes). The homogenates were centrifuged twice at 600 *g* for 10 min to remove nuclei and debris. The resulting supernatant was further centrifuged at 12,000 *g* for 15 min and the mitochondria, recovered in the pellets, were washed and resuspended in the same buffered-medium. The supernatant was used as the cytosolic fraction.

### Release of cytochrome *c* from mitochondria

*In vivo* release of cytochrome *c* from mitochondria into the cytoplasm of AVO-b-treated HL-60 cells was detected by probing the electrophoresed mitochondrial (30 μg) or cytosolic (50 μg) fractions, after blotting their proteins onto a PVDF membrane, with anti-cytochrome *c* antibody as previously described [35].

### Western blot analysis

AVO-treated HL-60 cells were harvested by centrifugation at 500 *g*, washed twice with PBS, and suspended in a lysis buffer containing 100 mM Hepes, pH 7.4, 10% sucrose, 10 mM DTT, 0.1% CHAPS, 150 mM NaCl, and protease inhibitors (1 mM PMSF and 1 μg/mL each leupeptin, aprotinin, and pepstain A). The cells were lysed by four repeated cycles of freeze/thawing (in dry ice/37°C water bath) and were then centrifuged at 4°C for 30 min at 14,000 *g*. The supernatant was collected and stored at -80°C or used immediately. The lysates were analyzed for total protein by a protein assay kit, based on the colorimetric reaction of Bradford protein assay (from Bio-Rad Laboratories Inc., CA, USA). Protein samples (50 μg/well) were separated on 10% or 12% SDS–polyacrylamide gels and electroblotted onto a PVDF membrane (Millipore Corp., MA, USA). Western blot analyses for caspases, cytochrome *c*, members of the Bcl-2 family proteins, Apaf-1 and XIAP were previously described in our publications [31,35]. To confirm equal loading of proteins in gels, the blots were also immunoprobed with a rabbit polyclonal antibody against the cytoskeletal protein α-tubulin or β-actin.

### Statistical analysis

Data presented are the means ± S.D. of results from a minimum of three independent experiments with similar patterns. Statistical analysis was performed using one-way ANOVA and Student’s t-test. A p < 0.05 value was considered statistically significant.

## Results

### AVO induces apoptosis in HL-60 cells

**Figure 1:**
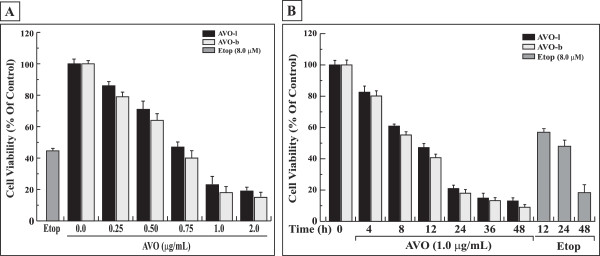
**AVO inhibits the growth of HL-60 cells in a dose- and time-dependent manner. (A)** HL-60 cells were treated with varying concentrations of AVO-l or AVO-b (0.0 to 2.0 μg/mL) for 24 h and the viability was analyzed by MTT assay as described in the methods. **(B)** HL-60 cells were incubated with 1.0 μg/mL of either AVO-l or AVO-b for different periods of time (0 to 48 h) and cell viability was assessed by the MTT assay. The control represents cells incubated under similar conditions in the absence of essential oil. The percentage of cell viability in each sample was expressed relative to untreated control, which was considered as a 100%. HL-60 cells were also treated with 8.0 μM etoposide under similar conditions as positive controls. The results shown represent the mean ± SD of three independent trials. Statistical analysis showed that all samples are significantly different (p < 0.05), when compared to untreated control without AVO or etoposide.

The cytotoxic effect of the essential oil (EO) extracted from both leaves and buds of *Artemisia vulgaris* was examined in HL-60 cells using MTT assays. The EO from both leaves (AVO-l) and buds (AVO-b) showed a substantial cytotoxicity in a dose- (Figure 1A) and time-dependent manner (Figure 1B). Interestingly, AVO-b showed 10-15% more cytotoxic effect than AVO-l in all the tested doses and time points. Accordingly, further experiments were performed to evaluate the effects of AVO on apoptosis and to identify the molecular mechanisms involved in its effect on HL-60 cells.

**Figure 2:**
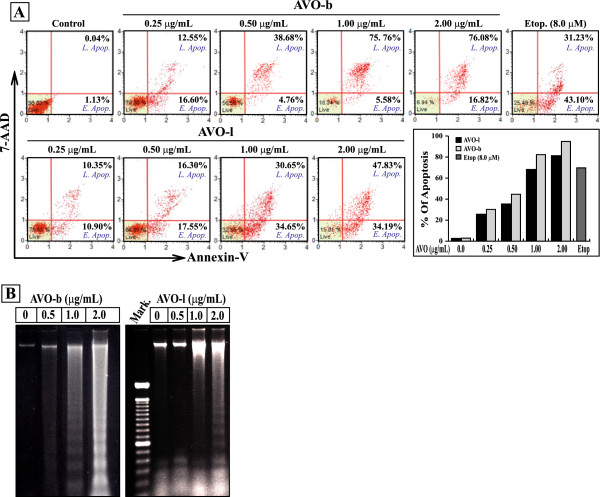
**The growth inhibitory effect of AVO on HL-60 cells is mediated by apoptosis. (A)** Cultured HL-60 cells were treated with varying concentration of AVO-b or AVO-l (0.0 to 2.0 μg/mL) an apoptosis was analyzed by a flow cytometer after staining with FITC-annexin-V and 7-AAD. The scattered blots showing the percentages of early and late apoptosis are indicated for one experiment. The graph represents the summary mean percentages of apoptosis (early and late apoptosis) of two independent experiments for each concentration of the oil. HL-60 cells were treated with 8.0 μM etoposide as a positive control. Statistical analysis showed that all samples are significantly different (p < 0.05), when compared to untreated controls. **(B)** Genomic DNA was extracted from HL-60 cells treated with the different concentrations of AVO-l or AVO-b, and applied to agarose gel electrophoresis. DNA fragmentation in the gel was visualized by UV light after staining with ethidium bromide. Mark, is a DNA ladder marker.

To determine whether the cytotoxic effect of AVO is associated with induction of apoptosis, HL-60 cells were treated with increasing concentrations of either AVO-b or AVO-l and then analyzed by a flow cytometry after staining with FITC-annexin-V/7-AAD. This method has been widely used to distinguish between normal cells (annexin-V^-^/7-AAD^-^), early apoptotic cells (annexin-V^+ve^/7-AAD^-ve^) and late apoptotic cells (annexin-V^+ve^/7-AAD^+ve^). As shown in Figure 2A, a dose-dependent increase in annexin-V^+ve^ cells was observed after treating with either AVO-b or AVO-l. Quantitative measurement of the percentages of apoptotic HL-60 pre-incubated with 0.25, 0.5, 1.0 and 2.0 μg/mL of AVO-l were 25.6, 35.4, 68.2 and 81.3%, while the same concentrations of AVO-b resulted in 30.2, 44.6, 82.3, and 94.8%, respectively. The average apoptosis rate produced by AVO-b was 20% higher than the one induced by AVO-l and, thus, coincided with the demonstrated effect by MTT assay. Furthermore, we examined the ability of AVO to trigger the formation of a typical internucleosomal DNA laddering pattern, as an indication for later apoptotic stages. As shown in Figure 2B, both essential oils from *A. vulgaris* induced DNA fragmentation in HL-60 cells in a dose-dependent manner. A clear DNA ladder fragmentation was detected after exposure of HL-60 cells to 1.0 μg/mL of AVO-b and 2.0 μg/ml of AVO-l for 24 h. Taken together, these findings indicate that the AVO-induced cell death in HL-60 cells is due to apoptotic rather than necrotic effect.

### Role of caspases in AVO-induced apoptosis in HL-60 cells

**Figure 3:**
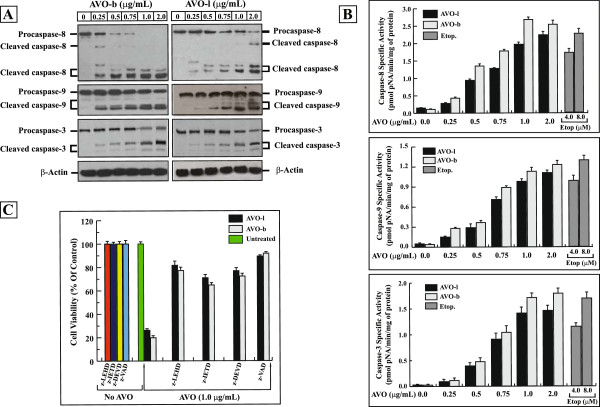
**The apoptotic effect of AVO is mediated by activation of caspase cascades. (A)** Whole cell extracts (50 μg), from HL-60 cells after treatment with varying concentrations of AVO-l or AVO-b for 24, were analyzed by Western blotting with antibodies against caspase-8 and -9 and caspase–3, respectively. The unprocessed forms of procaspase-8, -9 and -3, and their respective cleavage products of the active enzymes are indicated. The same membranes were also probed with an antibody against β-actin as a loading control. **(B)** Extracts (30 μg proteins) from cells treated with varying concentrations of AVO-l or AVO-b were analyzed for caspase-8, -9 and -3 catalytic activities using specific colorimetric tetrapeptide substrates. The specific enzyme activities, which represent the mean ± SD of three independent experiments, were measured as described in the methods. HL-60 cells were treated with 4.0 and 8.0 μM etoposide as positive controls. Statistical analysis showed that all samples are significantly different (p < 0.05), when compared to their untreated control. **(C)** HL-60 cells were incubated with either 35 μM of Caspase-8 inhibitor (z-IETD-fmk), caspase-9 inhibitor (z-LEHD-fmk), caspase-3 inhibitor (z-DEVD-fmk), or 50 μM of the general caspase inhibitor (z-VAD-fmk) for 6 h prior to addition of 1.0 μg/mL AVO-l or AVO-b for 24 h. After incubation, cell viability was assessed by the MTT assay. The cell viability for each sample treated with the specific caspase inhibitor was expressed as percentages relative to the parallel control cells incubated with the same caspase inhibitor alone, without the essential oil, which was considered 100%. The results shown represent the means ± SD of three independent experiments. Statistical analysis showed that all samples treated with caspase inhibitors and AVO are significantly different (p < 0.05), when compared to samples treated with the specific caspase inhibitor alone, AVO alone or untreated control.

Apoptosis induced by various cytotoxic agents is highly dependent on the activation of caspases, which play pivotal roles in cleaving specific target proteins [5]. To further characterize the AVO-triggered apoptosis, we examined whether treatments with the AVO-b and AVO-l lead to processing and subsequent activations of caspases in HL-60 cells. Therefore, cells were exposed to increasing concentrations of the EO for 24 h, and then analyzed for caspase processing by Western blotting (Figure 3A), and their enzymatic activation by colorimetric assays (Figure 3B). As shown in Figure 3A, the processing (activation) of initiator caspase-8 and -9 and the effector caspase-3 were demonstrated by the decrease in the levels of procaspase forms and the appearance of their corresponding cleaved bands after immunoblotting with their respective antibodies . The processing of these caspases increased after exposure of the HL-60 cells to the AVO-b and AVO-l in a dose-dependent manner, with maximum levels occurred after exposure to 2.0 μg/ml of AVO-b or AVO-l. The processing of caspases coincided with the demonstrated dose-dependent fragmentation of DNA (Figure 2B). Processing of these caspases was associated with their activation. As shown in Figure 3B, the AVO-l and AVO-b were found to trigger a dose-dependent increase in the specific activities of the initiator caspase-8 and -9 and the effector caspase-3 reaching a mean peak values (2.28 ± 0.12, 1.05 ± 0.08 and 1.41 ± 0.14 pmol *p*NA/min/mg protein, respectively) after incubating HL-60 cells with a concentration of 2.0 μg/mL of AVO-l for 24 h. Under similar experimental conditions, the AVO-b also induced the activations of these three caspases in a dose-dependent fashion, and reached a mean specific activity peak values at a concentration of 1.0 μg/mL of cultured cells for caspase-8 (2.68 ± 0.07 pmol pNA/min/mg protein) and 2.0 μg/mL for both caspase-9 and -3 (1.28 ± 0.09 and 1.83 ± 0.11 pmol pNA/min/mg protein, respectively). In all the tested doses, the AVO-b was able to induce a 20% to 35% more activation of these caspases than AVO-l. These data support the finding in Figures 1 and 2 that AVO-b is more toxic to cells than AVO-l. Interestingly, concentrations above 2.0 μg/ml of either oils did not show higher levels of caspases specific activities (data not shown).

**Figure 4:**
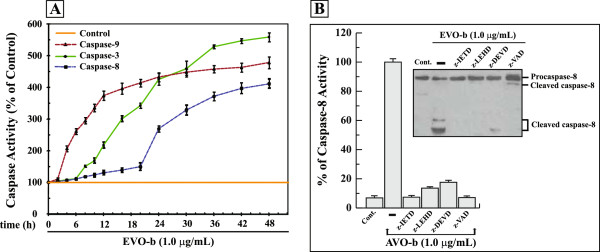
**AVO-b-induced processing and activation of caspase-8 in HL-60 cells requires activation of caspase-9 and -3. (A)** HL-60 cells were treated with 1.0 μg/mL AVO-b for different time intervals (0 to 48 h), and caspase-9 (▲), caspase-8 (■) and caspase-3 (●) activities were determined calorimetrically and expressed as relative percentages to the control untreated cells. The data shown represent the means ± SD of two independent trials for each time point. **(B)** AVO-b-induced caspase-8 activation in the presence and absence (-) of 35 μM specific caspase-8 inhibitor (z-IETD-fmk), caspase-9 inhibitor (z-LEHD-fmk), caspase-3 inhibitor (z-DEVD-fmk), or 50 μM of the general caspase inhibitor (z-VAD-fmk). HL-60 cells were incubated with each of the individual caspase inhibitor for 6 hours before the exposure to 1.0 μg/mL AVO-b for an additional 24 h. Caspase-8 activation was analyzed calorimetrically after addition of the substrate Ac-IETD-*p*NA (the graph) and by immunoblotting with a specific anti-caspase-8 antibody (the Western blot). The results shown represent the percentages of caspase-8 activity relative to the control cells treated with AVO-b alone (-). “Cont.”, represents caspase-8 activity in HL-60 of untreated cells. Results of caspase-8 activity represent the means ± SD of three indecent trials. Statistical analysis showed that all samples treated with specific caspase inhibitors and AVO-b are significantly different (p < 0.05), when compared to cells treated with AVO-b alone.

To further confirm the involvement of caspases in AVO-induced apoptosis, various specific caspase inhibitors, namely, z-LEHD-fmk (caspase-9 inhibitor), z-IETD-fmk (caspase-8 inhibitor), z-DEVD-fmk (caspase-3 inhibitor), and z-VAD-fmk (a general caspase inhibitor), were used at concentrations that completely blocked the activations of their corresponding caspases. HL-60 cells were incubated with the respective inhibitors for 6 hours prior to addition of the EO. As shown in Figure 3C, z-LEHD-fmk, z-IETD-fmk, z-DEVD-fmk and z-VAD-fmk all substantially suppressed the induced-cytotoxicity of AVO-l/AVO-b by 81.65%/77.14%, 70.93%/64.76%, 76.85%/72.33% and 89.30%/91.71%, respectively. Similarly, pre-incubating the cells with z-VAD-fmk completely inhibited the associated apoptosis changes induced by treating HL-60 cells with either AVO-l or AVO-b, such as the appearance of nnexin-V^+ve^ cells and DNA fragmentation (data not shown). These results imply that AVO-l and AVO-b induce apoptosis through a caspases-dependent pathway in HL-60 cells.To determine the signaling events involved in the AVO-induced activation of caspase cascades, we examined the effects of AVO-b on the kinetics of the activation of caspases-9, -8, and -3. As shown in Figure 4A, caspase-9 activation was detected at 4 h after oil treatment, whereas caspase-3 activation was evident at 8 h and caspase-8 activation was notable only at 16 h. The increased activities of caspase-9, -8, and -3 peaked at 48 h after exposure of the cells to the oil. These findings suggest that AVO signals activation of caspase-9 initially, which subsequently stimulates caspase-3 enzymatic activity, which then initiates caspase-8 activation to amplify the apoptosis cascade.

Caspase-8 activation can be triggered by a caspase-3-dependent feedback amplification loop of caspase cascades [37]. Therefore, we analyzed whether caspase-9 and caspase-3 activation is required for the activation of caspase-8 in AVO-b-treated HL-60 cells. As shown in Figure 4B, pre-incubation with z-LEHD-fmk (caspase-9 inhibitor) and z-DVED-fmk (caspase-3 inhibitor) markedly abolished the AVO-b-stimulated processing and activation of caspase-8. These results indicate the critical role caspae-9 and -3 in the AVO-induced apoptosis.

### Induction of mitochondrial death pathway by AVO in HL-60 cells

**Figure 5:**
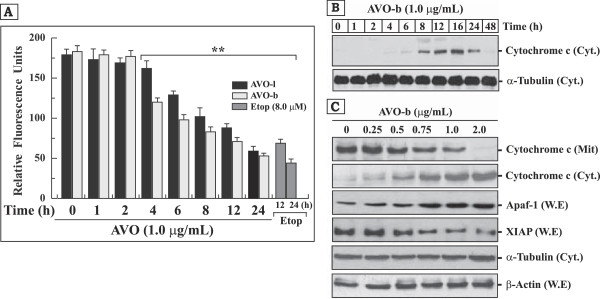
**AVO induces disruption of mitochondrial transmembrane potential (ΔΨ**_**m**_**), promotes the release cytochrome *c *to the cytoplasm and modulates the expression of Apaf-1 and XIAP proteins in HL-60 cells. (A)** HL-60 cells treated with 1.0 μg/ml AVO-l or AVO-b for different periods of time (0–24 h) and changes in mitochondrial membrane potential was monitored by a flourimetric analysis after addition of the fluorescent stain JC-1. Cells treated with 8.0 μM etoposide for 12 and 24 h were used as positive controls. The results shown represents the mean ± SD of three independent experiments. **Represents samples that are statistically different, when compared to their controls at 0 time or AVO untreated cells. **(B)** Western blot analysis of cytochrome *c* in the cytosolic fraction (50 μg) of HL-60 cells treated with AVO-b for different time points (0 to 48 h) as indicated at the top of each lane. **(C)** Western blot analysis of Apaf-1 and XIPA in whole cell extracts (W.E; 50 μg), and cytochrome *c* in cytosolic (Cyt.; 50 μg) and mitochondrial (Mit.; 30 μg) fractions obtained from HL-60 cells treated with varying concentrations of AVO-b (0.0–2.0 μg/mL). The same Western blots in both **(B)** and **(C)** for the cytosolic fractions and whole cell extracts were probed with antibodies to α-tubulin and β-actin, respectively, to ensure equal protein loading in the different lanes.

The activation of caspase-9 by AVO suggests that the mitochondrial apoptotic pathway is triggered in HL-60 cells. Mitochondria play an important role in cell death by changing its outer and inner membrane permeability and thus leading to cytochrome *c* release and caspases activation [38]. To explore whether the AVO-induced apoptosis occurs via the mitochondrial signaling pathway, we measured the effect of AVO-b and AVO-l on disruption of the mitochondrial membrane potential using the specific fluorescent dye JC-1. HL-60 cells treated with the AVO-b or AVO-l at a concentration of 1.0 μg/mL were found to demonstrate a loss of fluorescence intensity (red/green) with time (Figure 5A). A marked drop in the relative fluorescence was evident after 4 h incubation with the AVO-b, whereas the change in the relative fluorescence was noted after 6 h in cells treated with the AVO-l. These results indicate that both oils induce a loss of mitochondrial membrane potential.

Activation of the mitochondrial death pathway can also be identified by the release of mitochondrial cytochrome *c*. After cytochrome *c* is released from the mitochondria, it can bind to deoxyadenosine triphosphate (dATP) and Apaf-1, and activate caspase-9 and caspase-3 [7,8]. We, therefore, investigated the release of cytochrome *c* from the mitochondria into the cytosol by Western blotting. Cytosolic cytochrome *c* was detected by varying the exposure time (Figure 5B) and concentrations of AVO-b (Figure 5C) in HL-60 cultured cells, and the levels of cytochrome *c* that remained in the mitochondria was observed to decrease concomitantly (Figure 5C).

Cytochrome *c* release from mitochondria is a critical step in apoptosis, and earlier investigations had shown that ionizing radiation (IR) and etoposide induced the release of cytochrome *c* from mitochondria in two distinct stages [39]. At the early stage, low levels of cytochrome *c* are released from mitochondria and activate caspases-9 and -3. In contrast, the late stage cytochrome *c* release leads to a drastic loss of mitochondrial cytochrome *c* and is associated with a reduction of the ATP levels and mitochondrial transmembrane potential. After accumulation, cytochrome *c* is progressively degraded by caspase-like proteases [40]. Indeed, our immunoblotting analysis showed a small amount of earlier cytochrome *c* release after 4–6 h exposure to AVO-b. The amount of cytochrome *c* released to the cytosol increased reaching a peak at 16 h indicating the late stage of heavily loss of mitochondrial pool of this protein (Figure 5B). The gradual disappearance of cytochrome *c* from the cytosol at later hours (24–48 h) could be due to its degradation by caspase-like proteases [40]. The ability of AVO-b to release cytochrome *c* from mitochondria to the cytosol of HL-60 treated cells in concentration-dependent manner (Figure 5C) suggests that the compounds in this extract specifically target the mitochondrial pathway of apoptosis. AVO-l also induced a decrease of mitochondrial cytochrome *c* in a similar fashion to the AVO-b, but the amount of cytosolic cytochrome *c* that was induced by AVO-l was lesser than that induced by AVO-b and started to appear at 8 h after exposure to the oil (data not shown). This may be attributed to differences in the constituents or their concentrations in the two oils [23].

Cytosolic cytochrome *c* activates caspase-9 in the Apaf-1apoptosome, which subsequently triggers activation of the downstream executioner caspase-3 [7,8]. As shown in Figure 5C, levels of Apaf-1 were elevated after exposure of HL-60 cells to increasing concentrations of AVO-b, whereas the levels of the mitochondria-induced apoptosis inhibitor XIAP [41] decreased, and thus further supporting the involvement of the mitochondrial pathway in AVO-triggered apoptosis.

**Figure 6:**
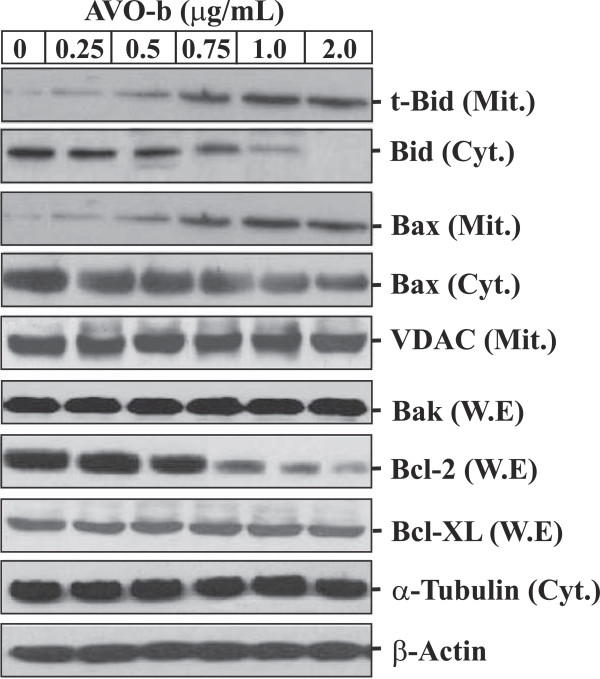
**AVO-b modulates the expression of Bcl-2 family proteins, and promotes translocation of Bax and t-Bid to the mitochondria of HL-60 cells in a dose-dependent manner.** Whole cell extracts (50 μg), mitochondrial (30 μg) and cytosolic (50 μg) fractions were prepared from HL-60 cells treated with AVO-b as described above. W.E were probed with antibodies against Bak, Bcl-2 and Bcl-xL proteins. Mit. fractions were immuneblotted with antibodies against t-Bid, Bax and VDAC, while Cyt. fractions were probed with antibodies for Bid and Bax. The same Western blots for the Cyt. fractions and W.E were probed with antibodies to α-tubulin and β-actin, respectively.

Since Bcl-2 family proteins are known to control a mitochondria-mediated apoptosis pathway by maintaining a balance between pro- and anti-apoptotic members [9], we examined the effects of AVO-b on the expression levels of Bcl-2 family proteins in HL-60 treated cells (Figure 6). Our results showed that AVO-b compounds decreased the cytosolic levels of pro-apoptotic Bid, but increased the levels of t-Bid in mitochondria. Furthermore, exposure of HL-60 to the oil increased the expression of Bax and its translocation to mitochondria in a dose-dependent manner (Figure 6). On the other hand, the amount of the anti-apoptotic protein Bcl-2 was observed to decrease dramatically after treatment with AVO-b (Figure 6). In contrast, the expression of proapoptotic protein, Bak, the anti-apoptotic Bcl-xL did not change in the presence of the oil. The expression of VDAC was also not affected by treating AVO-b. These findings suggest that AVO modulates the protein levels of Bid, Bax and Bcl-2, and this result in loss ΔΨ_m_ and the release of cytochrome *c* from mitochondria to the cytosol.

### AVO induces apoptosis in several cancer cell lines

**Figure 7:**
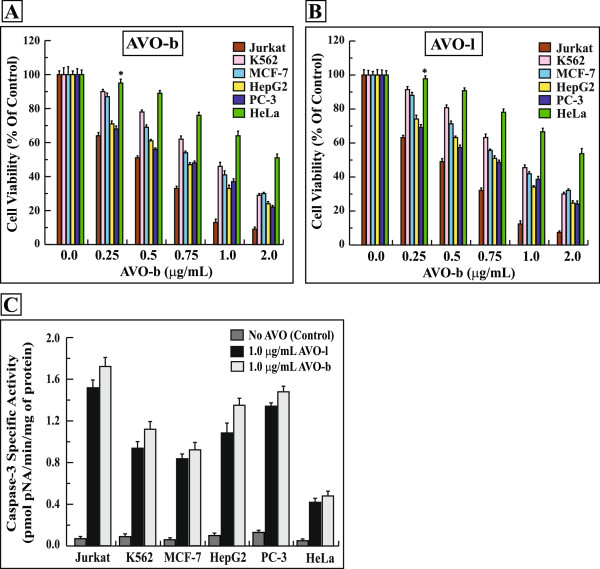
**AVO inhibits the growth of various cancer cell lines by inducing a caspase-dependent apoptosis.** Jurkat, K562, MCF-7, HepG2, PC-3 and HeLa cells were treated with varying concentrations (0.0-2.0 μg/mL) of AVO-b **(A)** or AVO-l **(B)** and the viability was analyzed by MTT assay. The data shown represent the mean ± SD of three independent experiments. **(C)** Extracts (30 μg proteins) from these cancer cells treated with 1.0 μg/mL of AVO-b or AVO-l were analyzed for caspase-3 catalytic activities. The specific enzyme activities, which represent the mean ± SD of three independent experiments, were measured as described in the methods. *p < 0.01 of statistically insignificant values, when compared to untreated samples.

In addition to the effect on HL-60 cells, we have also evaluated the potential apoptosis-mediated anticancer potency of AVO on different cancer cell lines. The AVO inhibited the growth of Jurkat, K562, MCF-7, HepG2, PC-3 and HeLa cells in a dose dependent manner of AVO-b (Figure 7A) and AVO-l (Figure 7B). In all the tested cell lines, AVO-b was more potent inhibitor of growth than AVO-l. The growth Inhibitory effect of AVO in the different cell lines was associated with activation of caspase-3 in a dose-dependent manner (Figure 7C). Similar to HL-60, these findings suggest that the antitumor activity of AVO is a caspase dependent.

The IC_50_ values for the AVO-b were 0.5 *μ*g/ml in Jurkat, 0.72-0.74 μg/mL in HepG2 and PC-3, 1.0 μg/mL in K562 and MCF-7 and 2.5 μg/mL in HeLa cells (Table 1). Interestingly, the IC_50_ for the normal human skin fibroblast (BJ cells), human epithelial kidney cells HEK-293 and the Chinese hamster lung fibroblast V79-4 were much higher than any of the above cancer cells (17.75 μg/mL and 13.24 μg/mL and 11.25 μg/mL of AVO-b, respectively), suggesting the selectivity of the oil compounds to inhibit tumor cells (Table 1).

**Table 1:** **IC**_**50 **_**values of AVO-b in different cell lines**

^**a**^**Cell line**	**Origin**	**IC50 AVO-b μg/mL**
Jurkat	Human Blood T-Cell	0.50
K562	Human Blood B-Cell	1.00
MCF-7	Human Breast	1.00
HepG2	Human Liver	0.72
PC-3	Human Prostate	0.74
HeLa	Human Cervical	2.50
BJ	human Skin	17.75
HEK-293	Human Kidney	13.24
V79-4	Chinese Hamster Lung	11.25

^a^The different cell lines were incubated with increasing concentrations of AVO-b (0.0 to 20.0 μg/mL) for 24 h. after incubations, the cell viability was assessed by MTT assay. The IC_50_ values were determined by using non-linear regression analysis of GraphPad Prism 5 software.

## Discussion

Despite the increasing understanding of the biological processes of cancer development, there is still a strong need for novel and effective pharmacological strategies for intervention with this disease [2]. Pharmacological agents that induce apoptosis might be effective against many cancers by inducing death in cancer cells [3,4]. One common and effective strategy for developing novel chemotherapeutics is the evaluation of natural compounds. In fact, a great number of clinically active drugs that are used in cancer therapy are either natural products or based on natural products. Established plant-derived therapeutics include vinblastine, vincristine, etoposide, teniposide, paclitaxel, doxetaxel, and camptothecin [42]. Plants from the genus *Artemisia* L. are phytochemically important due to their chemical and biological diversity. Species belonging *Artemisia* are widespread throughout the world and most popular in Chinese and other Asian countries traditional medicine for treatment of various diseases such as malaria, hepatitis, inflammation, cancer, and viral, bacterial and fungal infections [10,11]. Extracts from *Artemisia kulbadica*, *Artemisia diffusa*, *Artemisia sieberi*, *Artemisia santolina* and *Artemisia turanica* all showed cytotoxic effects on different cancer cell lines [43,44]. Recently, the methanolic extract from *Artemisia vulgaris* has been shown to substantially reduces the viability of the hepatocellular carcinoma cell line HepG2 [20]. The cytotoxic effect of this extract is suggested to be mediated by apoptosis. Extract from *A. vulgaris* have been also reported to induce apoptosis in prostate, breast and colon cancer cell lines [21] and sensitize MDA-MB-231 and MDA-MB-468 breast cancer cells to TRAIL [22]. However, neither the composition of active ingredients nor the detailed apoptotic mechanisms induced by the different extracts have been determined.

In this report, we show, for the first time, that low doses of the essential oil, contained in the leaves (AVO-l) and buds (AVO-b) of *A. vulgaris* L*.*, induce apoptosis in HL-60 and various other human cancer cell lines (Jurkat, K562, MCF-7, HepG2, PC-3 and HeLa), but lacks substantial cytotoxicity for normal non-malignant mammalian cells such as the human skin fibroblasts BJ and kidney epithelial cells HEK-293 or the Chinese hamster V79-4 lung cells at comparable low concentrations. This feature implies a promising potential of AVO compounds as chemotherapeutic for cancer treatment with a low risk of side effects, usually related to the unspecific cytotoxicity of many conventional cancer therapeutics [4]. AVO decreased cell viability after 24 h exposure in a dose-dependent manner, as assessed by MTT assay in HL-60 and the six other cancer cell lines (Jurkat, K562, MCF-7, HepG2, PC-3 and HeLa). Moreover, AVO caused cell death in the HL-60 cell line in a time-dependent manner, as determined by the same MTT assay. In general, the cytotoxicity assays of the volatile oil extracted from buds (AVO-b) showed 10% to 15% more effect than the oil extracted from leaves (AVO-l) of the plant. This may be due to differences in the constituents or their concentrations in the two oils [23].

Recent studies have suggested that various extracts and essential oil from different *Artemesia* species possess a chemopreventive potential that is mediated through apoptosis. The antiproliferative effect of chloroform extract from *Artemisia turanica* on HL-60 and K562 cancer cells is mediated by apoptosis, whereas the J774 normal cells have not been affected significantly by this extract [45]. Sesquiterpene lactones compounds derived from *Artemisia douglasiana* promote accumulation of DNA damage markers such as phosphorylated form of ATM and focal organization of γH2AX and 53BP1, therefore, triggering cell cycle arrest and apoptosis in HeLa, S3, MCF-7 and WI-38 cancer cells [46]. The marked killing effect of certain sesquiterpene lactones derivatives on cancer cells guided them to clinical trial studies [47]. In addition, it has been recently shown that the toxicity of *Artemisia annul* L. on cultured hepatocarcinoma cells SMMC-7721 is mainly mediated by apoptosis [48]. Essential oils of *Artemisia capillaris* and *Artemisia iwayomogi* have been reported to induce apoptosis in KB human oral epidermoid carcinoma cells via mitochondrial stress and caspase activation mediated by MAPK-stimulated signaling pathway [15].

Cell death may occur by many ways such as necrosis, autophagy, mitotic catastrophe, senescence and apoptosis [49]. For AVO-induced cell death, apoptosis seems to be way of how cancer cells die. This is supported by the following findings: first, AVO treated HL-60 cells showed the morphological aspects associated with early and late apoptotic events when assessed by flow cytometry after staining with FITC-Annexin-V/7-AAD. Second, AVO induced activation of caspase-3, -8, and -9 that typically mediate and execute apoptotic cell death. Third, AVO caused DNA laddering and fragmentation in apoptotic nuclei of HL-60 cells, a well-recognized process typically occurring during apoptosis [49]. Fourth, AVO caused a loss of the mitochondrial ΔΨ_m_ and evoked the release of cytochrome *c* into the cytoplasm in a dose- and time-dependent manner, a characteristic for numerous stimuli that cause apoptosis via the intrinsic pathway involving mitochondria [3,4]. Note that in contrast to cancer HL-60 cells, AVO up to 10 μg/mL failed to trigger processing of caspase-3, -8 and -9, fragmentation of genomic DNA, and loss of the ΔΨ_m_ in non-transformed BJ cells (data not shown), correlating to the moderate susceptibility of these cells to AVO-induced cell death. Finally, AVO failed to evoke apoptosis in HL-60 cells in the presence of caspase-3, caspase-8 caspase-9 or the general caspases inhibitors, implying that a select caspase-dependent pathway is required to evoke cell death by AVO.

Though the nature of the target of AVO that initializes apoptosis is unknown, our data suggest that AVO signals via the intrinsic pathway, rather than the death-receptor/FADD/caspase-8-mediated signaling route. AVO induced a loss of ΔΨ_m_, release of cytochrome *c* to the cytoplasm, increased the expression of Apaf-1, decreased the expression of XIAP (which inhibits caspase-9 and -3 activation), required caspase-9 for induction of cell death, activated effector caspase-3 and caused DNA fragmentation. Together, these events typically signal within the intrinsic pathway acting downstream the release of cytochrome *c* from mitochondria [50-52]. Our findings that substantial activation of caspase-9 occur earlier than caspase-8 activation, and that caspase-8 activation was inhibited in the presence of caspase-9 inhibitor in AVO-treated cells strongly suggest that the AVO-induced apoptosis occurs mainly through cytochrome *c*-dependent activation of caspase-9 and that activation of caspase-8 is a subsequent event to activation of the caspase cascade.

AVO also clearly caused processing of caspase-8, that usually occurs in response to ligation of death receptors such as CD95 or the TNFα receptor involving FADD [6]. Since active caspase-8 is capable of inducing the release of cytochrome *c* from mitochondria involving cleavage of Bid to t-Bid and its subsequent translocation to mitochondria [9], AVO could also affect mitochondria via such a route. Together, the intrinsic pathway involving mitochondrial events and caspase-9 virtually contributes to AVO-evoked cell death, whereas the extrinsic pathway involving death receptors, FADD and caspase-8 seems to amplify the apoptotic signal, through increasing the release of cytochrome *c* to the cytoplasm by cleaving Bid and its subsequent translocation to mitochondria [9].

Certainly, the elucidation of the pathways and the respective targets of AVO involved in apoptosis are of interest. Possible upstream routes and central candidates for mitochondria-mediated apoptosis that are targeted by typical cancer agents include the pro-apoptotic factor p53 and Bcl-2 family of proteins. The pro-apoptotic protein p53 plays a major role in cellular response to DNA damage, and when activated leads to cell cycle arrest and DNA repair or to apoptosis [26,53,54]. p53 protects cells against cancer transformation and, therefore, many apoptosis inducing chemotherapeutics depend on functional p53 [55]. Most of human cancers have either mutations or defects in the p53 pathway [53]. HL-60, PC3 and Jurkat cells that are deficient in functional p53 [53,54,56] were highly susceptible for AVO, suggesting that AVO induces apoptosis in a p53-independent manner.

The BH3-only proteins act as sentinels for cell stress, damage or infection, thereby initiating mitochondrial outer membrane permeabilization by oligomerization of Bax and/or Bak in the mitochondrial outer membrane forming channels that permit cytochrome *c* to escape from the mitochondria [57]. Since the BH3-only proteins target pro-apoptotic as well as anti-apoptotic Bcl-2 factors [58], an orchestrated interplay of these proteins is important for normal cell proliferation and cell death, whereas an influence on a select BH3-only protein may cause an imbalance of these processes. Our finding that AVO caused a decrease in the expression of Bcl-2 and increased the translocation of Bax and t-Bid to mitochondria strongly suggest that the oil-induced apoptosis involves modulating the expression of both the anti-apoptotic and pro-apoptotic members of the Bcl-2 family. AVO may also govern the function of a pro-apoptotic Bcl-2 member or, on the other hand, may suppress an anti-apoptotic family member. Alternatively, AVO may directly act on the mitochondria, hence leading to loss of the mitochondria ΔΨ_*m*_ and/or allowing cytochrome *c* to escape from this organelle. Current experiments in our lab address the proximal signaling steps in AVO-induced loss of ΔΨ_*m*_ and cytochrome *c* release.

## Conclusions

In this study, we have shown for the first time that the natural ingredients of *A. vulgaris* EO act as a strong and selective inducer of apoptosis in various cancer cells with lower cytotoxicity in normal non-transformed cells. Obviously, mitochondrial events are the determinants in AVO-induced cell death, and caspase-9 is a critical element in the transduction of the apoptotic signal, whereas the extrinsic pathway involving death receptors and caspase-8 is less important. We have recently identified 22 different components in the essential oil or *A. Vulgaris* L. [23]. Major components of the oil such as caryophyllene, alpha-zingiberene, borneol and ar-curcumene all have been reported to induce apoptosis [24-26]. However, very little is known in scientific research about the properties of *A. vulgaris* EO compounds and few studies have addressed their pharmacological actions. Therefore, it will be an exciting challenge to further characterize the pharmacology of *A. vulgaris* EO compounds and, in particular, their anticancer pharmacological actions.

## Abbreviations

EO: Essential oil; AVO: *Artemisia vulgaris* essential oil; AVO-l: *Artemisia vulgaris* leaves essential oil; AVO-b: *Artemisia vulgaris* buds essential oil; ΔΨ_m_: Difference in mitochondria transmembrane potential; Apaf-1: Apoptotic protease activating factor-1; XIAP: X-linked inhibitor of apoptosis protein; Bcl-2: B-cell lymphoma 2; Bcl-xL: B-cell lymphoma-extra-large; Bax: Bcl-2-associated X protein; Bak: Bcl-2 homologous antagonist killer; Bid: BH3 interacting-domain death agonist; t-Bid: Truncated Bid; VDAC: Voltage-dependent anion-selective channel; MTT: 4,5-dimethylthiazol-2yl)-2,5-diphenyltetrazolium bromide; JC-1: 5,5′,6,6′-tetrachloro-1,1′,3,3′-tetraethylbenzimidazolocarbocyanine iodide; z-LEHD-FMK: N-Benzyloxycarbonyl-Leu-Glu (OMe)-His-Asp (OMe)-fluoromethyl ketone; z-DEVD-FMK: N-Benzyloxycarbonyl-Asp (OMe)-Glu (OMe)-Val-Asp (OMe)-fluoromethyl ketone; z-VAD-FMK: N-Benzyloxycarbonyl-Val-Ala-Asp-fluoromethyl ketone; z-IETD-FMK: Methyl 5-[[1-[(5-fluoro-1-methoxy-1,4-dioxopentan-3-yl) amino]-3-hydroxy-1-oxobutan-2-yl] amino]-4-[[3-methyl-2-(phenylmethoxycarbonylamino) pentanoyl] amino]-5-oxopentanoate; Ac-LEHD-pNA: N-acetyl-Leu-Glu-His-Asp-*p*-nitroanilide; Ac-IETD-pNA: N-acetyl-Ile-Glu-Thr-Asp-*p*-nitroanilide; Ac-DEVD-*p*NA: N-acetyl-Asp-Glu-Val-Asp-*p*-nitroanilide; *p*NA: *p*-nitroanilide.

## Competing interests

The authors declare that they have no financial and/or non-financial competing interests.

## Authors’ contributions

AMS carried out apoptosis assays, DNA fragmentation, mitochondria membrane potential measurements, Western blots for cytochrome *c* and drafted the manuscript. AA performed Western blot for caspases and Bcl-2 family proteins. SAAR carried out MTT assays, several Western blots and tissue culture of the different cell lines. AN conducted the colorimetric caspase assays. ASA conceived the manuscript, organized between contributors and performed tissue culture. JDW prepared the essential oil from *A. vulgaris*, confirmed the composition of AVO-l and AVO-b, planned for the project with AMS and participated in drafting the manuscript. All authors have read and approved the final manuscript.

## Pre-publication history

The pre-publication history for this paper can be accessed here:

http://www.biomedcentral.com/1472-6882/14/226/prepub
